# A phase I trial of preoperative radiotherapy and capecitabine for locally advanced, potentially resectable rectal cancer

**DOI:** 10.1038/sj.bjc.6602106

**Published:** 2004-08-10

**Authors:** S Y K Ngan, M Michael, J Mackay, J McKendrick, T Leong, D Lim Joon, J R Zalcberg

**Affiliations:** 1 Division of Radiation Oncology, Peter MacCallum Cancer Centre, St Andrews Place, East Melbourne, Victoria 3002, Australia; 2 Division of Haematology and Medical Oncology, Peter MacCallum Cancer Centre, Melbourne, Victoria, Australia; 3 Division of Surgical Oncology, Peter MacCallum Cancer Centre, Melbourne, Victoria, Australia; 4 Department of Oncology, Box Hill Hospital, Melbourne, Victoria, Australia

**Keywords:** capecitabine, rectal cancer, preoperative radiotherapy, phase I trial

## Abstract

The purpose of the study was to determine the maximum-tolerated dose (MTD) of oral capecitabine, combined with concurrent, standard preoperative pelvic radiotherapy, when given twice daily, from Monday to Friday throughout the course of radiotherapy, for locally advanced potentially resectable rectal cancer. Maximum-tolerated dose was defined as the total (given in two equally divided doses) oral dose of capecitabine that caused treatment-related grade 3 or 4 toxicity in one-third or more of the patients treated. Radiotherapy involved 50.4 Gy given in 28 fractions in 5 weeks and 3 days. Eligible patients had a newly diagnosed clinical stage T3–4 N0–2 M0 rectal adenocarcinoma located within 12 cm of the anal verge suitable for curative resection. Surgery was performed 4–6 weeks from completion of preoperative chemoradiotherapy. In all, 28 patients were enrolled in the study at predefined dose levels: 850 mg m^−2^ day^−1^ (*n*=3), 1000 mg m^−2^ day^−1^ (*n*=6), 1250 mg m^−2^ day^−1^ (*n*=3), 1650 mg m^−2^ day^−1^ (*n*=3), 1800 mg m^−2^ day^−1^ (*n*=8) and 2000 mg m^−2^ day^−1^ (*n*=5). The mean age was 62.3 years (range: 33–80 years). Five patients were female and 23 male. The median distance of tumour from the anal verge was 6 cm (range: 1–11 cm). Endorectal ultrasound was performed in 93% of patients. A total of 26 patients (93%) had T3 disease and two patients had resectable T4 disease. Dose-limiting toxicity (DLT) developed in one patient at dose level 1000 mg m^−2^ day^−1^ (RTOG grade 3 cystitis). Two of the five patients at dose level 2000 mg m^−2^ day^−1^ had a total of three DLT (grade 3 perineal skin reaction, grade 3 diarrhoea and grade 3 dehydration). Dose escalation of capecitabine was ceased at 2000 mg m^−2^ day^−1^ after reaching MTD. None of the eight patients at dose level 1800 mg m^−2^ day^−1^ developed DLT. All except one patient underwent surgery. A total of 15 patients had the clinical T stage reduced by at least one stage in pathologic specimens. Five patients (19%) achieved a pathologic complete response. We conclude that the MTD of capecitabine was reached at a dose level of 2000 mg m^−2^ day^−1^, given as 1000 mg m^−2^ twice daily, from Monday to Friday throughout the course of preoperative pelvic irradiation of 50.4 Gy. For patients with resectable rectal cancer receiving concurrent, full dose radiotherapy, the recommended dose of capecitabine for further study is 1800 mg m^−2^ day^−1^ when given in this schedule.

Local recurrence after curative treatment remains a very significant cause of morbidity from carcinoma of rectum as the pelvic anatomy limits lateral resection margins (Rich *et al*, 1983; Chan *et al*, 1985; Quirke *et al*, 1986). Combined postoperative radiotherapy and chemotherapy reduces the rate of local recurrence and prolongs survival in patients with Dukes B2 and C rectal cancer (Thomas and Lindblad, 1988; Krook *et al*, 1991; O'Connell *et al*, 1994). As a result, adjuvant chemoradiation has been recommended as standard therapy for Dukes B2 and C rectal cancer (NIH Consensus Conference, 1990). Although a quality of life study has confirmed the value of postoperative adjuvant radiotherapy and chemotherapy, concern about treatment-related toxicity remains (Ooi *et al*, 1999).

Preoperative radiotherapy has the potential advantages of less acute and long-term toxicity (Sauer, 2003). Intra-abdominal adhesions are uncommon in the undisturbed peritoneal cavity, in which small bowel is freely mobile. Placing the patient in the prone treatment position allows the small intestine to move out of the radiation volume, thereby reducing the risk of radiation enteritis. In the preoperative setting, the local tissue environment is also more favourable with the tumour having a better blood supply, thus minimising the problem of hypoxia – an important cause of failure of radiotherapy. Another potential advantage is the increased chance of sphincter preservation in locally advanced rectal cancer as a result of tumour response (Crane *et al*, 2003), but it remains a controversial issue.

Although randomised trials have shown that short-course hypofractionated radiotherapy reduces local recurrence and improves survival (Swedish Rectal Cancer Trial, 1997; Camma *et al*, 2000; Kapiteijn *et al*, 2001), this approach has not been accepted worldwide as the standard preoperative approach. However, preoperative radiotherapy in conventional fractionation schedules, given concurrently with continuous infusion 5-fluorouracil (5FU), has been widely adopted as the preferred preoperative regimen, given the acceptable toxicity profile and efficacy when this regimen is given in the postoperative setting (O'Connell *et al*, 1994).

Infusional 5FU requires a central venous access line and an ambulatory delivery system. It is inconvenient to the patient and carries risks including venous thrombosis, infection and intravenous line migration. An effective oral delivery 5FU would overcome these problems.

Capecitabine is an oral fluoropyrimidine. Its efficacy as first-line treatment in metastatic colorectal cancers (MCRC) has been shown in randomised trials (Twelves, 2002). Its safety as an alternative to intravenous 5FU-based adjuvant therapy for colon cancer has been addressed (Scheithauer *et al*, 2003). Capecitabine achieves long-term inhibition of the target enzyme thymidylate synthase, and in this way mimics the continuous intravenous infusion of 5FU. It has the additional important theoretical advantage of the targeted production of 5FU in malignant cells via the enzyme thymidine phosphorylase (TP), which is overexpressed in tumour *vs* normal tissue (Miwa *et al*, 1998). In addition, radiotherapy is known to further upregulate TP, and in xenograft models, capecitabine and radiation have shown supra-additive activity (Sawada *et al*, 1999). Therefore, the combination of capecitabine with radiotherapy merits clinical investigation. Thus, a phase I trial of the combination of standard, preoperative radiotherapy and escalating doses of concurrent capecitabine was undertaken to assess the risk/benefit ratio.

## PATIENTS AND METHODS

The protocol, detailed patient information sheet and consent form were reviewed and approved by the human research ethics committees of participating centres. The trial was conducted in accordance with the Declaration of Helsinki, International Good Clinical Practice principles as described in the International Conference of Harmonisation (ICH) guidelines for Good Clinical Practice, as well as in accordance with all local ethical and regulatory requirements. Signed informed consent of patients was obtained before any trial-specific procedures were performed.

### Eligibility criteria

For this trial, eligible patients were required to have a pathologically documented adenocarcinoma of the rectum with the lower limit within 12 cm from the anal verge. Patients were 18–75 years old, although patients over the age of 75 years were eligible if in the opinion of the investigators they were fit and well for the purposes of the trial. Patients had to be considered suitable for curative resection by the participating surgeon at the time of study entry. Tumours were clinical stage T3–T4 tethered, or with radiological (ultrasound or CT) evidence of perirectal fat infiltration or nodal involvement. Examples of resectable T4 lesions were a rectovaginal or rectovesical fistula. The patient had to have a World Health Organisation (WHO) performance status of 0, 1 or 2. Patients with recurrent rectal cancer were not eligible.

The exclusion criteria were previous pelvic or abdominal radiotherapy or a scheduled dose of less than 50 Gy or treatment with high-dose per fraction (>2 Gy). Patients with evidence of metastatic disease, history of seizures, central nervous system disorders, clinically significant psychiatric disability, dementia, altered mental status or psychosis were not eligible. Patients with a history of severe and unexpected reaction to fluoropyrimidine therapy possibly related to DPD deficiency, or known hypersensitivity to 5FU were excluded. Patients with abnormal haematologic values (absolute neutrophil count <1.5 × 10^9^ l^−1^, platelet count <100 × 10^9^ l^−1^, Hb <9 g dl^−1^) or moderate to severe renal impairment, that is, creatinine clearance ⩽50 ml min^−1^, as calculated by the Crockroft Gault formula were not eligible. The use of blood transfusions to render patients eligible was allowed, but the use of growth factors to aid haematologic recovery was not allowed within 2 weeks before the start of or during the course of treatment. Pregnant or lactating women, women of child-bearing potential with either a positive pregnancy test at baseline or not using a reliable and appropriate contraceptive method were not eligible. Other exclusion criteria were autologous or allogeneic organ grafts, lack of physical integrity of the upper gastrointestinal tract, malabsorption syndromes, inability to tolerate or absorb oral medication or positive hepatitis B surface antigen, hepatitis C antibodies or human immunodeficiency virus type 1 or 2 antibodies.

### Trial design

This was a phase I trial of escalating doses of capecitabine combined with concurrent preoperative conventionally fractionated radiotherapy for locally advanced, potentially resectable rectal cancer. The primary objective was to determine the maximum-tolerated dose (MTD) of intermittent, twice daily oral capecitabine given with pelvic radiotherapy (in the schedule described) through the assessment of both subjective and objective adverse events reported during and up to 6 weeks after treatment. The secondary objectives were to determine the safety profile of the combination regimen and to describe any evidence of antitumour activity. The MTD was defined as the total oral dose (given in approximately two equally divided daily doses) of capecitabine when given intermittently (Monday to Friday) from the first till the last day of standard technique pelvic radiotherapy, which caused drug-related grade 3/4 toxicity in one-third or more of the patients treated (i.e. two or more in a six patient cohort).

Three patients were entered at each dose level. Subsequent dose levels were not opened until all three patients (or all additional patients where toxicity required more patients to be treated) had reached the 2-week point beyond the completion of concurrent chemotherapy and radiotherapy. If none of the three patients experienced a dose-limiting toxicity (DLT), then recruitment began at the next dose level. Otherwise, (a) when a DLT was seen in one patient out of the three patients, three additional patients were recruited on this dose level. If a DLT was observed in only one of the six patients, then recruitment began at the next dose level. If DLTs were observed in two or more of the six patients, no further dose escalation was to take place. (b) If two or more patients of the first three patients on a dose level had DLTs, no further dose escalation was to take place. The MTD was defined at that dose level. DLT was defined as grade 3 or 4 lower gastrointestinal or genitourinary toxicity (RTOG acute toxicity criteria), or perineal skin toxicity or any other grade 4 toxicity (RTOG acute toxicity criteria or NCIC Toxicity Criteria). A delay of >8 weeks before surgery for toxicity reasons after completing concurrent therapy was also considered a DLT. If the cohort at the starting dose level experienced toxicity defined as DLT, the dose was reduced to the preceding dose level, being two-thirds of the initial level until the treatment was considered to be tolerable.

### Trial treatments

Pelvic radiotherapy was given with a megavoltage machine (6–18 MeV) using a three- or four-field technique. The patient was treated in the prone position. A belly board and other methods were used to minimise treatment-related toxicity. Computerised dosimetry was routinely performed. The total radiation dose was 50.4 Gy in 1.8 Gy per fraction per day, 5 days per week. The first 45 Gy was given to the pelvic field and 5.4 Gy in three fractions were given to the reduced field. The total duration of treatment was 5 weeks and 3 days. The planning target volume for the pelvic field was: upper border at the L5/S1 junction, inferior border 3 cm below the primary tumour or at the inferior aspect of the obturator foramina, whichever was the most inferior, lateral border 1.5 cm lateral to the widest bony margin of the true pelvic side wall, anterior border 2 cm from the anterior boundary of the mesorectum and posterior border a minimum of 1 cm behind the anterior bony sacral margin. For the reduced field, a 2 cm margin was given to the site of gross disease. All radiation fields were treated daily, Monday to Friday. Verification port films were performed weekly.

Radiotherapy was temporarily interrupted at the investigator's discretion if there was grade 3 or 4 toxicity. The decision to interrupt treatment was taken only after discussion with the principal investigators. Radiotherapy was to recommence once toxicity had decreased to grade 2 or lower. If recovery of toxicity to grade 2 or less had not occurred after a 2-week delay, the treatment was ceased.

The planned escalating dose levels of capecitabine were 850, 1000, 1250, 1650, 1800, 2000 and 2500 mg m^−2^ day^−1^. Three patients were planned for each cohort. The cohort was expanded according to the previously described schedule. Capecitabine was given twice daily, Monday to Friday. The first daily dose of capecitabine was administered approximately 2 h±30 min before radiotherapy, at the same time every day, throughout the entire period of radiation. Capecitabine and radiotherapy treatment were commenced on the same day and were ceased together at completion of the preoperative therapy. The whole duration was 5 weeks and 3 working days (28 days). The total daily dose was divided into two approximately equal lots and administered separately approximately 12 h apart and within 30 min after the ingestion of food. Surgery (using total mesorectal excision) was performed after a rest period of 4–7 weeks from completion of the preoperative chemoradiation. In patients receiving low anterior resections, defunctioning ileostomy or colostomy was recommended.

### Dose modifications for toxicity

Treatment was continued without modification for grade 1 toxicity. If a patient experienced any grade 2 toxicity and the toxicity was considered to be mainly due to capecitabine treatment, drug administration was withheld until the grade 2 toxicity resolved to grade 0–1 before being restarted at the same dose with prophylactic treatment (for nausea, diarrhoea, hand–foot syndrome) where necessary and possible. If grade 2 toxicity recurred, capecitabine treatment was withheld until the toxicity resolved to grade 0–1 and the capecitabine treatment was restarted at the preceding dose level. In cases in which toxicity recurred at the starting dose level, capecitabine treatment was discontinued for the rest of the course of radiotherapy. In each of these circumstances, radiotherapy was not affected unless the described toxicity worsened after discontinuing capecitabine. If a patient experienced any grade 2 toxicity, which was mainly cutaneous, radiotherapy and capecitabine treatment were continued. If a patient experienced any grade 3 or 4 toxicity, with the exception of alopecia, which was considered mainly due to capecitabine treatment, both capecitabine and radiotherapy treatment were discontinued. Radiotherapy was recommenced without capecitabine for the remaining course of treatment once the toxicity had improved to grade 0–2. Capecitabine treatment was discontinued for any grade 3 or 4 haematological toxicity.

### Monitoring for safety

Assessments before treatment included demographic data, medical history, physical examination, haematology, renal and liver function tests, CEA, pregnancy test (if appropriate), and chest X-ray, ECG, abdominal and pelvic CT, endorectal ultrasound and PET scan. Routine assessments, performed prior to the start of treatment, weekly thereafter for 6 weeks and every second week up to 6 weeks after the last radiotherapy administration included vital signs, physical measurements, haematology, blood chemistry, urinalysis and monitoring of adverse events.

### Statistical considerations

This trial aimed to identify the MTD of intermittent twice daily oral capecitabine in combination with concurrent, standard radiotherapy. An escalating dose design was employed, in which a maximum of 12 patients could be entered at each dose level. The MTD was defined to be the first dose that caused a DLT (grade 3/4 toxicity – see prior section) in one-third or more of the patients treated. These prespecified rules were based on clinical and ethical considerations, rather than on statistical methods. The analyses of the primary and secondary efficacy variables comprised simple descriptive statistics, summary tables, plots and patient listings. The descriptive statistics included the mean, standard deviation, standard error, minimum and maximum for continuous variables. The median was used as the measure of central tendency for any time-to-failure end points. Counts and percentages were provided for discrete variables. The data were analysed using SAS version 8.2.

## RESULTS

### Patient characteristics

In all, 28 patients were enrolled in the trial. The mean age was 62.3 years, with a range of 33–80 years. Of these, 27 of the patients were Caucasians and one was Asian. Five patients were female and 23 were male. The WHO performance status of patients at the time of screening was mainly level 1 (54%) and level 2 (43%). The median distance of the lower border of the tumour to the anal verge was 6 cm (range: 1–11 cm). Rectal ultrasound examinations were performed in 93% of patients. A total of 26 patients (93%) had T3 disease and two patients had resectable T4 disease. The clinical stages of disease before treatment are listed in Table 1

**Table 1:** Pretreatment clinical stage

**Clinical stage**	
T3N0	13 (46)
T3N1	13 (46)
T4N0	1 (4)
T4N1	1 (4)

.

### Dose level

The dose levels of the patients accrued into this trial were: 850 mg m^−2^ day^−1^ (*n*=3), 1000 mg m^−2^ day^−1^ (*n*=6), 1250 mg m^−2^ day^−1^ (*n*=3), 1650 mg m^−2^ day^−1^ (*n*=3), 1800 mg m^−2^ day^−1^ (*n*=8) and 2000 mg m^−2^ day^−1^ (*n*=5).

### Adverse events

Four DLTs occurred in three patients – one of six patients at a dose of 1000 mg m^−2^ day^−1^ and two of five patients at a dose of 2000 mg m^−2^ day^−1^. Dose escalation was stopped at 2000 mg m^−2^ day^−1^ – the protocol-defined MTD of capecitabine in this study. After reaching MTD at 2000 mg m^−2^ day^−1^, five additional patients were treated at 1800 mg m^−2^ day^−1^ giving the 1800 mg m^−2^ day^−1^ cohort a total of eight patients. The patient with a DLT at the dose level of 1000 mg m^−2^ day^−1^ developed grade 3 urological toxicity at day 12 of treatment, which resolved slowly after discontinuation of all treatment. One patient at dose level 2000 mg m^−2^ day^−1^ developed a DLT with grade 3 perineal skin toxicity requiring opioid analgesics at day 14 of treatment. A second patient at this dose level developed two DLTs – grade 3 diarrhoea and grade 3 dehydration on day 22.

One patient developed pulmonary embolism after completion of the preoperative therapy. The most common adverse events were gastrointestinal disorders with 15 patients (54%) reporting at least one such event, skin and subcutaneous tissue disorders in nine patients (32%) reporting at least one such event, general disorders such as fatigue or pain in eight patients (23%) and renal and urinary disorders in six patients (21%). Hand–foot syndrome occurred in 11% of patients: grade 1, 3.6% and grade 2, 7.1%. There was no grade 2 or 3 neutropenia or thrombocytopenia encountered. Table 2

**Table 2:** Acute toxicities (worst grade)

**Toxicities**	**Grade**	**850 mg m^−2^ day^−1^**	**1000 mg m^−2^ day^−1^**	**1250 mg m^−2^ day^−1^**	**1650 mg m^−2^ day^−1^**	**1800 mg m^−2^ day^−1^**	**2000 mg m^−2^ day^−1^**
*Haemoglobin*							
	0	1	4	2	2	5	4
	1	0	0	0	1	2	1
	2	2	2	1	0	1	0
							
*Neutrophils*							
	0	3	6	3	3	7	5
	1	0	0	0	0	1	0
							
*Platelets*							
	0	3	5	2	2	6	5
	1	0	1	1	1	2	0
							
*Genitourinary*							
	0	1	2	3	1	2	2
	1	2	2	0	1	6	2
	2	0	1	0	1	0	1
	3	0	1	0	0	0	0
							
*Lower GI*							
	0	0	2	0	1	2	0
	1	2	1	3	2	5	1
	2	1	3	0	0	1	3
	3	0	0	0	0	0	1
							
*Skin*							
	0	0	0	0	2	1	1
	1	1	1	3	1	6	2
	2	2	5	0	0	1	1
	3	0	0	0	0	0	1
							
*Hand-foot syndrome*							
	0	3	5	3	3	6	5
	1	0	0	0	0	1	0
	2	0	1	0	0	1	0

shows the acute toxicity according to dose levels.

### Surgical morbidity

Surgical resection of the rectal cancer was performed after preoperative chemoradiation in 27 patients. One patient refused to have surgery after achieving a clinical complete response. Sphincter sparing surgery was performed in 44% (12 patients). Abdominoperineal resection was performed in 52% (14 patients). Proctocolectomy was performed in one patient. There was one postoperative death. The patient developed an acute myocardial infarct and died 4 days after operation. The postoperative complications are listed in Table 3

**Table 3:** Postoperative complications

**Complications**	**Number of events**
Wound complications	5
Infection	5
Urinary tract complications	3
Nausea and vomiting	3
Abdominal distension and ileus	2
Anaemia	2
Thrombosis	1
Haemorrhage	1
Rectal bleeding	1
Anal ulcer	1
Abdominal pain	1
Cardiogenic shock	1
Cerebral infarction	1
Leukopenia	1

*Note*: Surgical procedures *n*=27, as one patient refused surgery. Each patient may have more than one event.

.

### Antitumour activity

Out of a total of 27 patients, 15 had a lower T stage (by at least one stage) in the resected specimen compared with pretreatment clinical stage after preoperative chemoradiation. Five patients (19%) achieved a pathologic complete response (no evidence of disease in the resected specimen). Table 4

**Table 4:** Comparison of pretreatment clinical T stage and pathological T stage after preoperative chemoradiation

	**Pathological T stage**						
**Clinical T stage**	**0**	**1**	**2**	**3**	**4**	***X***	**Total**
3	**5**	**0**	**8**	12	0	1^a^	26
4	**0**	**1**	**0**	**1**	0	0	2
							
Total	5	1	8	13	0	1	28

*Note*: Bold numerals represent downstaging by at least one stage.

a Patient refused surgery.

shows the correlation between the pretreatment clinical stage and the postsurgery pathologic stage.

## DISCUSSION

In advanced colorectal cancer, capecitabine is generally administered in an intermittent schedule of 1250 mg m^−2^ twice daily (2500 mg m^−2^ day^−1^) for 2 weeks followed by a 1-week treatment break. However, combined with radiotherapy, a continuous regimen seemed to be a hypothetically more attractive schedule, mimicking the postoperative adjuvant setting, in which the optimal approach to the treatment of resected T3–4/N0–2 rectal cancer includes radiotherapy combined with concurrent, continuous infusion 5FU (O'Connell *et al*, 1994). Furthermore, in nonrandomised trials of preoperative radiotherapy combined with continuous infusion 5FU, a pathologic complete response rate of 16–29%, low toxicity profile and no obvious increase in surgical morbidity have been reported (Rich *et al*, 1995; Ngan *et al*, 2001).

Infusional 5FU requires an ambulatory infusion device and continuous intravenous access. It carries risks of infection, venous thrombosis, intravenous line blockage and is inconvenient to patients. Capecitabine, an effective oral agent commonly used in MCRC, avoids all of these problems. In this phase I trial, the schedule of capecitabine was designed to simulate infusional 5FU to maximise radiosensitisation and cytotoxicity. Conventional, fractionated radiotherapy was given 5 days per week (Monday to Friday) and capecitabine was only given on the days of radiotherapy. The advantage of a ‘drug-free’ period of 2 days per week not only was thought to be more convenient but may have increased the dose intensity and hence radiosensitisation of chemotherapy.

In this trial, dose escalation of capecitabine was discontinued at the MTD of 2000 mg m^−2^ day^−1^ with two of five patients having developed a DLT. The three DLTs that occurred in these two patients were perineal cutaneous reactions, diarrhoea and dehydration. Overall, hand–foot syndrome was uncommon with grade 1 in one patient and grade 2 in two patients out of the total of 28 patients. The haematological toxicity of this regimen was also low with no grade 3 toxicity. Combined with concurrent preoperative conventionally fractionated radiation, a dose of capecitabine of 1800 mg m^−2^ day^−1^ was recommended for further study for locally advanced resectable rectal cancer. None of the eight patients at this dose level (1800 mg m^−2^ day^−1^) experienced a DLT.

In contrast, in a German trial in which the majority of patients (all with rectal cancer) were treated postoperatively and capecitabine was given 7 days a week throughout the course of pelvic radiotherapy (Dunst *et al*, 2002), two of the six patients treated with capecitabine at a dose of 2000 mg m^−2^ day^−1^ experienced grade 3 hand–foot syndrome. Dose escalation was stopped at 2000 and 1650 mg m^−2^ day^−1^ was the recommended dose for further evaluation. In a similar phase I trial from Greece (Souglakos *et al*, 2003), in which capecitabine was given continuously throughout the period of radiotherapy (in this study given postoperatively to all patients), three of the six patients developed a DLT, including one with grade 3 hand–foot syndrome and dose escalation was ceased at a capecitabine dose of 1700 mg m^−2^ day^−1^. In this trial, grade 1 hand–foot syndrome was noted in three out of 10 patients and grade 2 in three out of 10 patients. A Korean group has also reported their experience of oral capecitabine and concurrent radiation for rectal cancer treating 45 patients with clinical T3/T4 or node-positive rectal cancer preoperatively to 50.4 Gy (Kim *et al*, 2002). The chemotherapy schedule used in this study consisted of two cycles of the intermittent schedule (14 days of capecitabine 1650 mg m^−2^ day^−1^ and leucovorin 20 mg m^−2^ day^−1^ followed by a 7-day rest). Grade 3 hand–foot syndrome occurred in 7% of patients (grade 1, 31%, grade 2, 9% of patients). The other grade 3 toxicities included fatigue in 4%, diarrhoea in 4% and radiation dermatitis in 2% of patients.

Our study only included patients with T3 or resectable T4 rectal cancer. The clinical stage of the tumour was confirmed in the majority of cases (93%) with endorectal ultrasound. All except one patient underwent resection after preoperative therapy, providing a better opportunity than in previous studies to evaluate the antitumour activity of capecitabine combined with concurrent, preoperative radiotherapy. Pathologic complete response was noted in five of the 27 evaluable patients (19%). A total of 15 patients (56%) showed downstaging of the pretreatment clinical T stage by at least one stage compared to the pathologic T stage. This result was similar to a previous Australasian phase II trial with continuous infusion 5FU using similar radiation protocols and selection criteria (Ngan *et al*, 2001). In that trial, the pathologic complete response rate was 16% and downstaging of the pretreatment clinical T stage by at least one stage compared to the pathologic T stage was seen in 54% of patients.

In the German phase I trial, only 10 patients underwent preoperative chemoradiation, and of these, pathologic complete response was observed in one patient. In the phase I trial from Greece, antitumour activity could not be assessed because all patients were treated postoperatively. In the Korean study, 38 of the 45 patients received definitive surgery. Two patients only had a transanal excision and five patients refused surgery after completing the preoperative chemoradiation. The pathologic complete response rate for the group of patients who underwent definitive surgery was 31% (12 of 38). However, endorectal ultrasound was only performed in 18% of patients so that the extent of downstaging could not be accurately assessed.

Our trial also provided an opportunity for a formal assessment of the surgical morbidity from preoperative radiotherapy and concurrent capecitabine. The surgical morbidity was comparable with the Australasian preoperative radiotherapy trial with infusional 5FU. Surgical morbidity data were not presented for the 10 patients treated preoperatively in the German trial. In the Korean study, the postoperative complications listed were neurogenic bladder (one patient), intestinal obstruction (two patients including one patient who required surgery), anastomotic leakage requiring surgery (one patient) and rectovaginal fistula requiring surgery (one patient). Postoperative complications of our trial are summarised in Table 3.

## CONCLUSION

We conclude that the MTD of capecitabine was reached at a dose level of 2000 mg m^−2^ day^−1^, given as 1000 mg m^−2^ twice daily, from Monday to Friday throughout the course of preoperative pelvic irradiation of 50.4 Gy. For patients with resectable rectal cancer receiving concurrent, full dose radiotherapy, the recommended dose of capecitabine for further study is 1800 mg m^−2^ day^−1^ when given in this schedule.
